# Mutating for Good: DNA Damage Responses During Somatic Hypermutation

**DOI:** 10.3389/fimmu.2019.00438

**Published:** 2019-03-12

**Authors:** Bas Pilzecker, Heinz Jacobs

**Affiliations:** Division of Tumor Biology & Immunology, The Netherlands Cancer Institute, Amsterdam, Netherlands

**Keywords:** abasic site, base excision repair, cytosine deamination, DNA damage tolerance (DDT), non-canonical mismatch repair (ncMMR), translesion synthesis (TLS)

## Abstract

Somatic hypermutation (SHM) of immunoglobulin (*Ig*) genes plays a key role in antibody mediated immunity. SHM in B cells provides the molecular basis for affinity maturation of antibodies. In this way SHM is key in optimizing antibody dependent immune responses. SHM is initiated by targeting the Activation-Induced Cytidine Deaminase (AID) to rearranged V(D)J and switch regions of *Ig* genes. The mutation rate of this programmed mutagenesis is ~10^−3^ base pairs per generation, a million-fold higher than the non-AID targeted genome of B cells. AID is a processive enzyme that binds single-stranded DNA and deaminates cytosines in DNA. Cytosine deamination generates highly mutagenic deoxy-uracil (U) in the DNA of both strands of the *Ig* loci. Mutagenic processing of the U by the DNA damage response generates the entire spectrum of base substitutions characterizing SHM at and around the initial U lesion. Starting from the U as a primary lesion, currently five mutagenic DNA damage response pathways have been identified in generating a well-defined SHM spectrum of C/G transitions, C/G transversions, and A/T mutations around this initial lesion. These pathways include (1) replication opposite template U generates transitions at C/G, (2) UNG2-dependent translesion synthesis (TLS) generates transversions at C/G, (3) a hybrid pathway comprising non-canonical mismatch repair (ncMMR) and UNG2-dependent TLS generates transversions at C/G, (4) ncMMR generates mutations at A/T, and (5) UNG2- and PCNA Ubiquitination (PCNA-Ub)-dependent mutations at A/T. Furthermore, specific strand-biases of SHM spectra arise as a consequence of a biased AID targeting, ncMMR, and anti-mutagenic repriming. Here, we review mammalian SHM with special focus on the mutagenic DNA damage response pathways involved in processing AID induced Us, the origin of characteristic strand biases, and relevance of the cell cycle.

## Introduction

**Figure 1:**
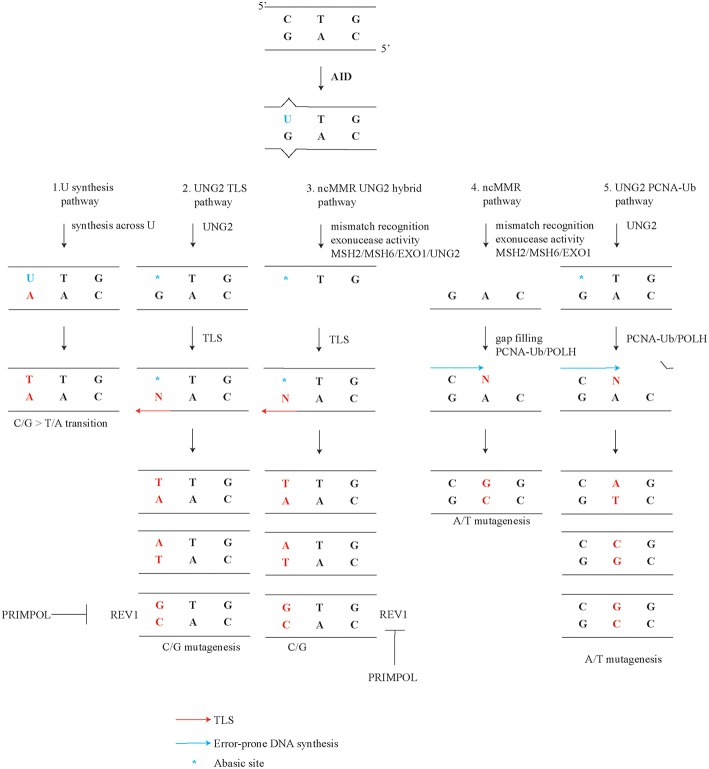
Mutagenic pathways of SHM. Deamination of C by AID during SHM leads to a specific mutagenic spectrum. The creation of the full SHM spectrum depends on (1) replication opposite template U instructs a template T and generates transitions at C/G. (2) UNG2 dependent TLS generates C/G transversions. UNG2 converts a U into an abasic site. As abasic sites are non-instructive, TLS opposite these sites generates both transitions and transversions. (3) Hybrid pathway between non-canonical mismatch repair (ncMMR) and UNG2 dependent TLS generates transversions at C/G. (4) ncMMR generates the majority of mutations at A/T. (5) UNG2- and PCNA Ubiquitination (PCNA-Ub)- dependent mutations at A/T. This non-canonical long-patch BER pathway generates a minor but significant subset of A/T mutations (~8%).

Somatic hypermutation (SHM) occurs in antigen-activated germinal center B cells and contributes to antibody affinity maturation (1–8). Class switch recombination (CSR) involves a deletional rearrangement process within the immunoglobulin (*Ig*) heavy constant region, which enables B cells to switch the isotype of the clonotypic antibody, adapt its effector functions, and alter its tissue distribution (9–11). SHM and CSR are both initiated by the Activation-Induced Cytidine Deaminase (AID), which has a preference for targeting single-stranded DNA of rearranged *Ig* genes (5, 12–17). SHM correlates with transcription and promotor proximal transcriptionally active regions in immunoglobulin genes appear to be preferred targets of AID (18–22). AID deaminates cytosines in the DNA into deoxy-uracil (U) during the G1 phase of the cell cycle, though Us and abasic sites may persist into S phase (23–25). AID activity and error-prone processing of the resulting U increases the mutation rate in Ig genes by an estimated six orders of magnitude, specifically from ~10^−9^ to ~10^−3^ mutations per base pair per division (26–28). AID preferentially targets WRCY motif (W = A/T, R = A/G, and Y = C/T). Another hotspot is the W*A* motif, though this motif is not targeted by AID (29–32). Five modes of mutagenic U processing are thought to be involved in generating the well-defined mutational spectrum of somatically mutated *Ig* genes (Figure 1) (1, 8, 33–37). SHM profiles comprise both transversions, where a pyrimidine base (C or T) is substituted by a purine base (A or G) or the reverse, and transitions, where a pyrimidine base (C or T) or a purine base (A or G) is replaced by the same class of base. In this review we discuss these modes, which are (1) replication opposite template U generates transitions at C/G, (2) UNG2 dependent translesion synthesis (TLS) generates transversions at C/G, (3) a hybrid pathway between non-canonical mismatch repair (ncMMR) and UNG2 dependent TLS generates transversions at C/G, (4) ncMMR generates mutations at A/T, and (5) UNG2 and PCNA Ubiquitination (PCNA-Ub) dependent mutations at A/T. Furthermore, the origin of tandem mutations, the characteristic strand biases, and cell cycle dependency of SHM are discussed. This review focuses on reports in mammalian systems. Findings in the DT40 lymphoma cell line have been reviewed elsewhere (38, 39).

## DNA Damage Responses Involved in Somatic Hypermutation

The DNA damage response plays a key role in SHM and takes advantage of defined components involved in DNA repair and DNA damage tolerance (40, 41). Here we will explain how these DNA damage repair and DNA damage tolerance pathways are repurposed to establish SHM pathways.

Normally, base modifications including Us in the DNA are efficiently recognized and repaired by the base excision repair (BER) pathway (42–44). To excise U from DNA backbone BER can use either one of four glycosylases, namely UNG, TDG, SMUG1, and MDB4 (45). However, only UNG was implicated in SHM (46). SMUG1 is downregulated in hypermutating B cells, though upon overexpression is able to rescue *Ung*-deficiency (47). Furthermore, SMUG1 was only found to have an effect on SHM and CSR in an *Ung*-deficient background (48). Initiating BER of Us, Uracil-(N)-glycosylase UNG recognizes and excises the irregular base from the sugar-phosphate backbone, creating an abasic site. At abasic sites the sugar phosphate backbone is intact. To further repair the abasic site, an incision is made beside the abasic site by AP-lyase APEX1 or APEX2 to yield a 3′ hydroxyl adjacent and a 5′ deoxyribose-phosphate (dRP). APEX2 is active in germinal center B cells, although it has a weaker AP-nuclease activity compared to APEX1 (49–51). Subsequently, POLB processes the abasic site and fills in the single nucleotide gap. Finally, ligases LIG1 or LIG3 seal the 3′ hydroxyl and 5′ phosphate groups. This pathway is known as short patch BER. In contrast, long patch BER involves strand displacement synthesis by replicative polymerase POLD. In contrast to POLB, POLD requires the homotrimeric DNA clamp PCNA as a processivity factor. After the incision by an AP-lyase, strand displacement synthesis by POLD is followed by removal of the displaced single-stranded DNA flap by FEN1, and finally ligation by LIG1 completes long patch BER.

In addition to processing of U by BER, the U can also be recognized as a U-G mismatch and the mismatch can be repaired by mismatch repair (MMR) (52, 53). During replication, DNA polymerase errors can give rise to mismatches, which are recognized by the mismatch recognition complex MSH2/MSH6 (52, 54). MSH2/MSH6 is tethered to the replication fork in order to detect mismatches and initiate mismatch repair. Next, the endonuclease complex PMS2 and MLH1 can make an incision 5′ of the mismatch. This nick serves as an entry point for exonuclease EXO1, which by means of its 5′ to 3′ exonuclease activity creates a single-stranded gap, which normally is filled up by replicative polymerases (55–57). Next to the canonical replication-associated MMR, an alternative ncMMR pathway that is predominantly active during G1 has been identified (52, 53, 58). ncMMR is thought to introduce mutations during SHM. During ncMMR, MSH2/MSH6 recognizes the AID induced U-G mismatch. The incision is made by the PMS2 and M LH1 endonuclease complex (53, 58). Subsequently, exonuclease EXO1 generates a single-stranded gap, though EXO1 does not necessarily remove the U containing strand. Next, the gap is filled in an error-prone manner by the translesion synthesis (TLS) polymerase POLH, a member of the Y-family of TLS DNA polymerases which lack proof-read activity (52, 53, 58). Effective recruitment of POLH to the single-stranded gap depends on monoubiquitination of the DNA clamp PCNA at lysine residue K164 (PCNA-Ub) (59–61). By tethering DNA polymerases to the template, the homo-trimeric DNA clamp PCNA serves as a critical processivity factor of DNA polymerases.

To establish somatic mutations, TLS as part of the DNA damage tolerance system plays an important role during SHM. During TLS, specialized polymerases can continue DNA replication or DNA repair synthesis by inserting a nucleotide opposite of the lesion. During replication, TLS may occur at the replication fork to allow replication to continue or during the filling in of post replicative gaps. In this manner TLS is thought to prevent prolonged fork stalling or even a fork collapse (62, 63). When the replication fork or repair synthesis is stalled by an abasic site, PCNA is monoubiquitinated by ubiquitin ligase complex RAD6/RAD18 (59, 60, 64–66). The formation of PCNA-Ub is a key step in the recruitment of damage tolerant, error-prone TLS polymerases, where the PCNA interacting peptide (PIP) warrants specificity and the UBM or UBZ motif in TLS polymerases increase the affinity to PCNA. In addition, REV1 exerts an PCNA-K164 ubiquitination independent function by recruiting other TLS polymerases via its C-terminal region (67–69).

An alternative DNA damage tolerance mode involves repriming behind the fork stalling lesion by PRIMPOL (70–73). PRIMPOL activity is thought to be restricted to the leading strand while the replicative primase POLα primes continuously on the lagging strand (73, 74).

## Replication Opposite Deoxy-Uracil Generates C/G Transitions

After C deamination by AID, DNA synthesis by any known DNA polymerase across the template U creates a C/G > T/A transition (Figure 1 point 1). If not recognized and processed by BER or MMR, the U in the DNA template will because of its close similarity to a T instruct the insertion of an A opposite the U (34, 75). In line, in the absence of UNG2 and MSH2 or MSH6, Us remain and cannot be shunted into other mutagenic pathways (see below). Consequently, C/G transitions were found almost exclusively in this setting. The resulting SHM profile is considered as the DNA footprint of AID activity (34, 76).

## UNG2 Dependent Translesion Synthesis Creates C/G Transversions

When a U is processed by UNG, an abasic site is generated. As abasic sites are non-instructive, replication opposite this lesion generates both C/G transversions and transitions (Figure 1, point 2) (36, 46, 77). A strong decrease of C/G transversions is observed in *Ung* deficient mice, however C/G transitions are increased, suggesting that abasic sites are key intermediates in the generation of C/G transversions (36, 46). Apparently, when Us are no longer processed into abasic sites, the increased number of Us leads to increased C/G transitions, as described above. There are two different splice variants produced from the *Ung* gene, mitochondrial localized UNG1 and nuclear localized UNG2 (78). As expected, only the nuclear isoform UNG2 was found relevant for SHM (79, 80).

POLB has a central function in short patch BER. At present, the role of POLB during SHM remains controversial. *Ex vivo* analyzed B cells from transplanted fetal liver HSC and progenitors deficient for *Polb* did not affect SHM (81). In contrast, another study found *Polb*-deficiency to mildly suppress SHM and CSR (82). The different results may be explained by a variation in the methods. The latter study has isolated and cultured the B cells for 4 days before the analysis, while the first performed analysis immediately after harvesting germinal centers.

As abasic sites are non-instructive and stalling entities to replicative DNA polymerases, specialized TLS polymerases are recruited to bypass this lesion. The TLS polymerase REV1 was found to be able to tolerate abasic sites (83, 84). The structure of its active site only allows REV1 to insert dCMP (85, 86). As such, REV1 is considered a dCMP transferase rather than a genuine polymerase. Next to its transferase capacity, REV1 has a BRCT and a C-terminal domain; the latter can recruit other Y-family members POLH, POLI, POLK (63). The inactivation of *Rev1* selectively prevents C/G to G/C transversions, in line with the dCMP transferase activity of REV1 (37, 87). The N-terminal BRCT domain of REV1 is involved in binding PCNA and does not affect SHM (88, 89). Further studies have shown that the catalytic domain of REV1 is key for C/G > G/C transversions (90, 91). In addition, in the presence of a catalytically inactive REV1 the TLS polymerase POLH appears involved in the generation of C/G > G/C transversions, though a *Polh* single mutant does not affect C/G > G/C transversions (91). It seems that the TLS recruitment function of REV1 plays a very limited role in SHM, as the SHM profile is similar in the REV1 catalytic dead mutant compared to the full knock-out.

To date, it remains unknown which polymerases are involved in G/C > T/A transversions, though in DT40 chicken lymphoma cell lines POLD3 as subunit of replicative polymerase delta has been suggested (92). This finding awaits corroboration in the mammalian system.

## Hybrid Pathway of Non-Canonical Mismatch Repair and UNG2 Dependent Translesion Synthesis Generates C/G Transversions

Interestingly, about half of all C/G transversions depend on the UNG2 and ncMMR hybrid pathway (Figure 1, point 3) (36, 37). In both the ncMMR dependent and independent arm, REV1 creates C/G > G/C transversions (37). This raises the question regarding the difference between C/G transversions relying only on UNG2 and those relying on both UNG2 and ncMMR. It has been suggested that C/G transversions in the AID hot spot AGCW depend on UNG2 alone, while C/G transversions outside this motif rely both on UNG2 and ncMMR (93). The authors propose that the ncMMR dependent and independent C/G transversions relate to the status of the cell cycle. How ncMMR and UNG2 cooperate to introduce C/G transversions remains largely unaddressed. We proposed that ncMMR either creates single-stranded DNA substrate for AID, or additional Us and abasic were already present before excision. Consequently, Us are modified by UNG2 into abasic sites to generate C/G transversions (36). The U on single-stranded DNA can be processed by UNG2 leading to C/G transversions. UNG2 is around 1.7-fold more effective on single-stranded DNA as compared to double-stranded DNA and therefore the MMR-generated single-stranded gap may provide a preferred UNG2 substrate (94). Alternatively, there may be a mutagenic repair pathway involving both UNG2 and ncMMR. Biochemical studies indicated that such a pathway indeed exists and demonstrated that UNG2 is involved in resolving U-G mismatches in cooperation with ncMMR (95). As UNG2 itself cannot provide the nick, APEX2 instead can nick the DNA downstream of UNG2 during abasic site processing in germinal center B cells, as initially proposed (51). Further studies should reveal which of the above sources of abasic templates contribute to the generation of MSH2/UNG2 dependent G/C transversions and the exact interplay between UNG2 and ncMMR.

## Mutagenic Non-Canonical Mismatch Repair Generates A/T Mutations

Cytosine deamination by AID generates a U-G mismatch, which can be repaired by ncMMR (52). During SHM, more than ninety percent of all A/T mutations depends on ncMMR (Figure 1, point 4). Recognition of the U-G mismatch requires the heterodimer MSH2/MSH6. The inactivation of *Msh2* or *Msh6* lead to impaired A/T mutagenesis (36, 75, 96, 97). Consistent with the single nucleotide mismatches generated by AID, the alternative mismatch recognition complex MSH2/MSH3 is not involved in A/T mutagenesis during SHM as it recognizes only long insertion/deletion loops and mismatches involving multiple bases (96, 97). Unexpectedly, endonuclease complex PMS2/MLH1, which is involved in ncMMR by making the incision for exonuclease EXO1, has a very small to no effect on A/T mutagenesis (98–100). However, when *Pms2* and *Ung* defective alleles are combined, the number of A/T mutations was found reduced to 50%, suggesting that both PMS2 and UNG2 are involved in making the incision for the entry of EXO1 during ncMMR (24). After recognition of the mismatch, exonuclease EXO1 is key in generating single-stranded DNA patches (101). These gaps can be filled in by TLS polymerase POLH in an error-prone manner (52, 102, 103). POLH is a highly error-prone polymerase with an *in vitro* error-rate of 10^−1^ to 10^−2^ mutations per base pair (104). Therefore, one would expect that it contributes to all mutation types during SHM. However, POLH has been shown to be preferentially error-prone at template TW motifs *in vitro*, explaining the contribution of POLH to A/T mutagenesis (105, 106). Interestingly, *in vitro* still one fourth of POLH induced mutations are C/G mutations, although this is not reflected in *Polh*-deficient mouse models (102, 103, 107). Another polymerase that does not have the same preference of POLH in mutating WA likely fills in the gap. Orthologs of POLH such as other members of the Y-family of polymerases REV1, POLK, and POLI may fill in the gap in absence of POLH. Indeed, the closest ortholog of POLH, POLK does contribute to A/T mutagenesis in absence of POLH (108–110).

Effective recruitment of POLH to the EXO-1 generated single-stranded DNA is mediated by PCNA-Ub during SHM (61, 103). Hereafter, POLH fills the gap in an error-prone PCNA-Ub dependent manner (61, 102, 103, 111). PCNA K164 is ubiquitinated by the RAD6/RAD18 ubiquitin ligase E2/E3 complex (59, 112). Remarkably, a *Rad18* defect had little to no effect on PCNA-Ub dependent A/T mutagenesis during SHM, as PCNA-Ub was decreased but still present (113). This is likely related to the existence of redundant E3-ligases, such as CRL4^Cdt2^ or RNF8 (114–116).

Generally PCNA ubiquitination is associated with replication stalling, the question how PCNA is ubiquitinated during ncMMR during G1 remains. Like during S phase, this may also be mediated by RPA coated single strand which recruit RAD6/RAD18.

In conclusion, ncMMR is key in generating the vast majority of A/T mutations during SHM.

## UNG2 and PCNA ubiquitination Dependent A/T Mutagenesis

**Figure 2:**
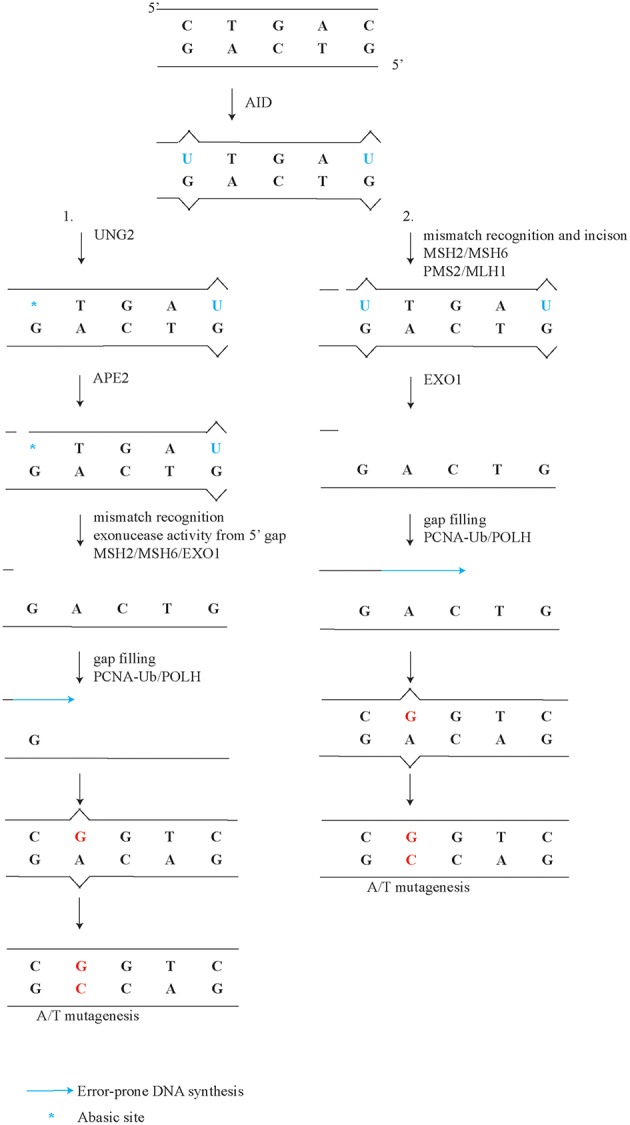
Detailed model of ncMMR in A/T mutagenesis. After U induction by AID, UNG2 processes U into an abasic site. In this more detailed model of ncMMR there are to arm on ncMMR, (1) UNG2 and APEX2 provide the incision for EXO1. EXO1 requires MSH2/6 and a 5' gap to the mismatch to be generate single-stranded DNA. PCNA-Ub recruits POLH, which can fill in the single-stranded DNA gap. (2) MSH2/MSH6 complex recognizes mismatch and activate the PMS2/MLH1 complex to make the incision. EXO1 creates singles stranded DNA gap, which is filled in by POLH.

A minor but significant subset of A/T mutations of about eight percent, is generated independently of ncMMR, although does depend on UNG2 and PCNA-Ub (Figure 1, point 5) (36, 117). Comparison of the mutation spectra from *Ung*^−/−^ and *Pcna*^*K164R*/*K164R*^ single mutant to *Ung*^−/−^*;Pcna*^*K164R*/*K164R*^ double mutant germinal center B cells revealed a further reduction of A/T mutations (36). This observation suggests that long-patch BER involving PCNA-Ub also contributes to A/T mutagenesis downstream of UNG2. Apparently, this non-canonical long patch BER has a minor but significant contribution to the generation of A/T mutations. This finding is in line with the observation that in B cells U-G mismatches and U-A base pairs are both mainly repaired by short patch BER (79). During both long-patch as well as short patch BER, APEX proteins are involved in the repair of abasic sites. Surprisingly, ubiquitously expressed APEX1 is downregulated while APEX2 is up-regulated in germinal center B cells (50, 51). Furthermore, *Apex2*-deficient B cells show a reduction of A/T mutagenesis (118, 119). Our reanalysis of the mutation frequency instead of percentages revealed a 60-80% percent reduction of A/T mutagenesis (Supplemental Table 1). Our reanalysis using mutation frequency instead of percentage of mutations revealed that C/G transitions and transversions were also decreased, in both datasets. The general reduction of all mutation types in *Apex2*-deficient mice is in line with the conclusion drawn in Sabouri *et al*. The discrepancy stresses the importance of determining mutation profiles with frequencies instead of percentages. Since long patch BER only generates a minor proportion of A/T mutagenesis, APEX2 is likely involved in the ncMMR pathway, for example by making incisions in the DNA which can be used by EXO1, as suggested previously (50, 51, 119) (Figure 2). Indeed, EXO1 is activated by a 5′ incision, a mismatch, and MSH2/MSH6 (120). In line, *in vitro* analysis of MMR and BER activity on a U-G mismatch containing plasmid demonstrated that an UNG2 dependent nick can be processed by EXO1 and MSH2/6 to effectuate MMR, independently of MLH1/PMS2 (95).

In summary, during SHM UNG2 and APEX2 contribute to the generation of a minor and a major part of A/T mutations, respectively. The prime role of APEX2 in A/T mutagenesis may be to provide the nick for both ncMMR and long patch BER.

## Tandem Mutations

Tandem mutations are two mutations in neighboring bases and around 5% of all mutations are tandem mutations (121). These may arise through independent mutagenic events in the neighboring bases. However, analysis of SHM profiles revealed that tandem mutations are found more frequently than expected by chance (121, 122). Furthermore, a part of all tandem mutations rely on the presence of MSH2 or MSH6, POLζ (REV3/REV7 complex), and POLI. Remarkably, both *Poli-* and POLζ subunit *Rev3-*deficient B cells displayed a decrease in tandem mutations (121, 122). The mutation load in POLζ subunit *Rev3-*defective B cells was lower. However, this may be due to an impaired proliferation rate in POLζ subunit *Rev3-*defective B cells (123). It is reasoned that POLI fills the gap that is generated by EXO1 during ncMMR in an error prone manner. As POLI can generate mismatches but not extend efficiently from those, the mismatch provided by POLI is likely to be extended by POLζ which subsequently generates the second mutation. POLH is also involved in ncMMR however, *Polh*-deficiency has no effect on tandem mutations (121, 122). These data suggested that during ncMMR predominantly POLH, but also POLI is involved in gap filling.

## Mutational Strand Biases of SHM

### AID Targeting and C/G Transition Bias

**Figure 3:**
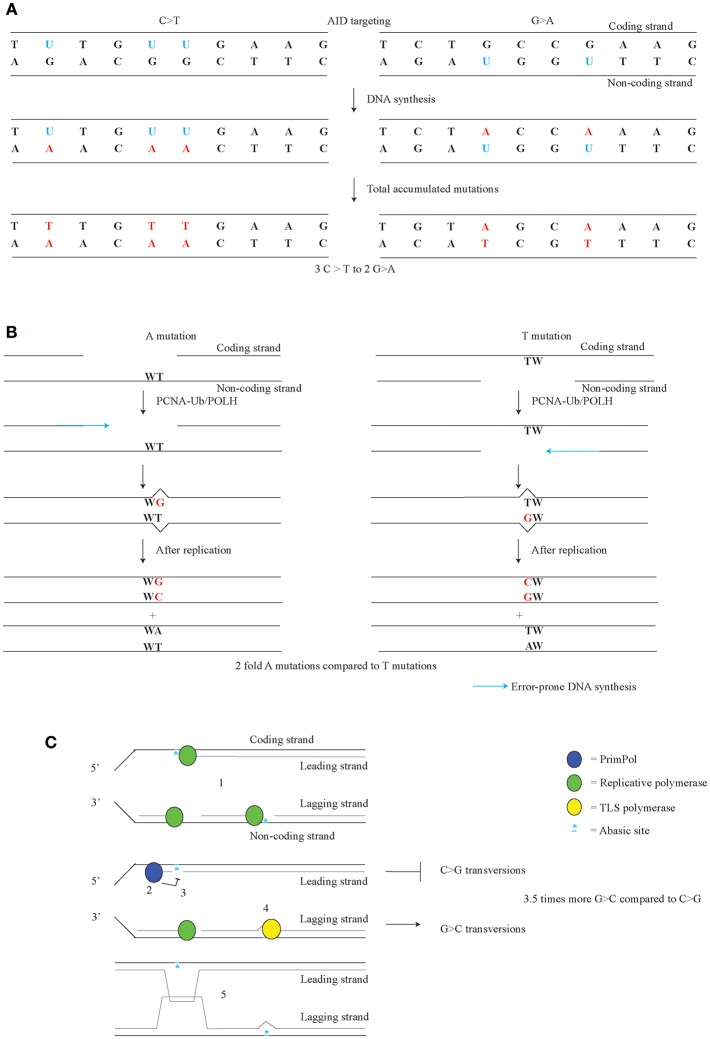
Strand-biases in SHM. **(A)** AID targeting with a preference for the coding strand leads to a C/G transition strand bias. Us on the coding strand lead to C>T transitions, while Us on the non-coding strand lead to a G>A transitions. **(B)** During error-prone mismatch repair, the MSH2/MSH6 complex recognizes the U-G mismatch, after which APEX2 or PMS2 provide the incisions for EXO1. POLH is especially error-prone on template TW. Therefore, the orientation of the gap made by EXO1 likely governs the A/T bias. **(C)** Replicative forks can be stalled on both leading and lagging strand by AID dependent abasic sites (1). After priming on the lagging strand, a replicative polymerase resumes DNA synthesis. PRIMPOL establishes G>C over C>G transversion bias found in *Jh4* intron of the *Igh* gene, likely though anti-mutagenic activity on the leading strand of replication. PRIMPOL restarts by repriming after stalled DNA synthesis (2) and prevents TLS (3). On the lagging strand, TLS opposite of the abasic site leads to G>C mutations (4). PRIMPOL activity likely activates a homology driven error-free pathway such as template switching to prevent mutagenesis (5). **(C)** adapted from Pilzecker et al. (73).

AID targeting occurs on both the coding and the non-coding strand of *Ig* loci. Differential targeting of AID to these strands has been implicated with the C/G transition bias observed in *Msh2;Ung* double mutant, as well as *Msh6;Ung* and *Pcna*^*K164R*^*;Ung* double mutant mice (34, 36, 75, 76). This C/G transition bias consists of the 1.5-fold higher number of C>T over G>A in the coding strand. After nucleotide correction, 60% of all C/G transitions arise on the coding strand and 40% on the non-coding strand (Figure 3A). This difference was considered to represent the AID targeting bias. Indeed as shown through measuring U content in the DNA, the amount of AID dependent U in the switch region are also 60% of Us are on the coding strand and 40% on the non-coding strand (124). AID is thought to target the single-stranded DNA of a transcription bubble of the coding strand, which is consistent with the C>T over G>A transition bias (17, 125). AID can also target DNA in DNA/RNA hybrids, G-structures, and supercoiled DNA which can all be found in transcribed genes (126–128). Differential distribution of these structures may contribute to the C/G transition bias.

### A/T Mutation Bias

Another strand bias found in SHM is the A/T bias, where in the coding strand A mutation are 2-fold more frequent than T mutations (Figure 3B) (33, 129, 130). As A/T mutagenesis is largely dependent on ncMMR pathway, it is likely that the A/T bias involves the ncMMR pathway as well. During ncMMR, EXO1 creates single-stranded gap to remove the mismatch. Error-prone filling of the gap by POLH is likely the cause of the A mutations at template TW (131). As POLH is especially error-prone at TW templates giving rise to WA hotspots, for A mutations to arise, the non-coding strand is used as template by POLH, whereas for T mutations, the coding strand is used as template by POLH. This means that the coding strand contains the gaps more frequently than the non-coding strand. The difference in gap formation, suggests that the U containing strand is removed, and that the AID targeting bias co-determines the A/T bias. However, in ncMMR the U containing strand was found not to be targeted specifically when the U-G mismatch is repaired (53).

In order to gain more insight into the A/T bias, a transgene containing a stretch of A and T with a C or a G in the middle was made (132). The analysis showed that the C in the coding strand leads to an increase in A/T mutagenesis in the surrounding of the C. Whereas, a G in the coding strand leads to the suppression of A/T mutagenesis. The downstream mutation bias could be suppressed by impairing *Msh2*. This suggests that ncMMR is needed to induce the mutation bias, though the study has a limited amount of mutations analyzed. Strangely, MMR component PMS2 seems to counterbalance to the A/T bias, even though PMS2 does not affect the number of A/T mutations. *Pms2*-defective mice show an increased A/T bias, due to both an increase of A mutations and a decrease of T mutations (24, 133). The authors hypothesize about the existence of a MLH1/PSM2 dependent pathway and an UNG/APEX2 dependent mismatch repair pathway. According to this model, the MLH1/PMS2 mismatch pathway has no strand bias, but UNG/APEX2 dependent pathway does. The actual strand bias is proposed to be the result of averaging the amount of bias of both pathways. When the MLH1/PMS2 pathway is impaired, the increased activity of the APEX2 dependent pathway could lead to an increased strand bias. This model suggests that the location of the U dictates the A/T bias. Hereafter, UNG2 and APEX2 cooperate to provide the incision on the U containing strand which is needed for EXO1 activity. As there are more Us found on the coding strand, there will be more EXO1 dependent single-stranded gaps on the coding strand, which lead to A mutations (Figure 3B) (124). This notion fits with the direction of the AID targeting bias and the A/T bias. Accordingly, one expects that an *Apex2-* or *Ung-*defect, would lead to a decreased strand bias. However, analysis of *Ung*-defective mice and our reanalysis of *Apex2* knock-out mice fail to demonstrate a decrease in the A/T bias (24, 118, 119) (Supplemental Table 1).

**Figure 4:**
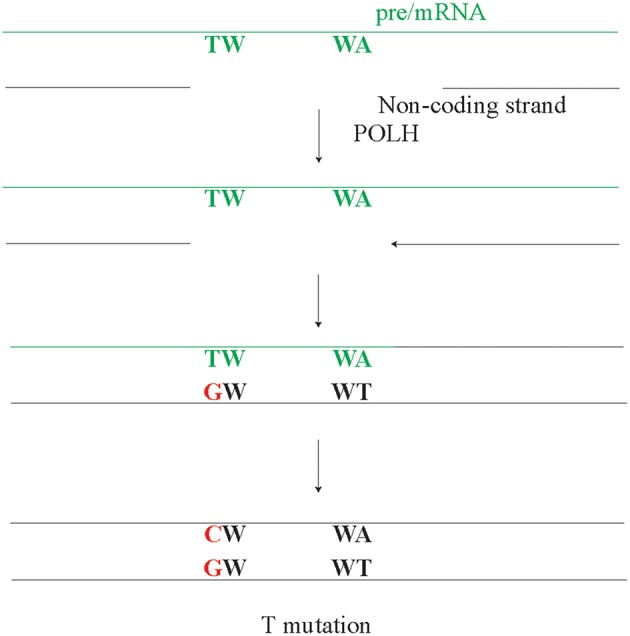
The potential but unlikely role of pre/mRNA in A/T mutagenesis. As the pre/mRNA is copied from the non-coding strand, it can only act as template for repair synthesis on the non-coding strand. After the pre/mRNA is copied from the non-coding strand, a gap can arise in the DNA-RNA hybrid on the non-coding DNA strand. As POLH has reverse transcriptase activity, this gap will be filled in an error-prone manner by POLH. However, if this potential mechanism or any other mechanism using the cDNA as an intermediate would be a dominant mode, a higher rate of T mutations compared to A mutations is expected, which directly contrasts the observed A/T bias.

Another model suggests that the A/T mutations arise though reverse transcriptase activity (134, 135). In line, POLH has been identified as DNA polymerase, RNA polymerase, and a reverse transcriptase (136–138). In fact, the reverse transcriptase activity of POLH has been implicated in A/T mutagenesis. However, potential POLH reverse transcriptase activity using the pre/mRNA as a template and the observed A/T strand bias are opposing each other, i.e., predict a higher amount of T rather than A mutations (Figure 4). The pre/mRNA is unlikely the source of A/T mutagenesis, unless it involves RNA editing at A preferentially in WA motifs. A POLH mutation leading to a POLH with an RNA or DNA specificity may provide the ultimate test, regarding the role of reverse transcription in SHM. However, involvement of RNA editing in A/T mutagenesis seems unlikely as A/T mutagenesis fully depends on AID and ncMMR, both of which target DNA.

In conclusion, the A/T bias involves ncMMR activity and arises through POLH using the non-coding strand as template more frequently.

### G>C Over C>G Transversion Bias

An additional bias found in *Jh4* intronic region in SHM is the 3.5-fold higher frequency of G>C over C>G mutation bias (73). C>G and G>C mutations arise from abasic sites, which suggests that there is either and unequal number of abasic sites on the coding and non-coding strand, or there is a difference of anti- or pro-mutagenic DNA damage responses on the coding and non-coding strand. The G>C over C>G bias is governed by PRIMPOL, as *Primpol*-deficiency was found to increase C>G transversions to the level of G>C transversions (Figure 3C). Apparently, an anti-mutagenic activity of PRIMPOL prohibits C>G transversions, which suggests that PRIMPOL exerts strand-biased anti-mutagenic activity at abasic sites. The dominant origin of replication in the *Igh* locus in B cells lies near the 3′ regulatory region enhancer (139–141). Therefore, most C>G mutations are likely to arise from abasic sites on the leading strand, whereas most G>C mutations arise from abasic sites on the lagging strand. Apparently, PRIMPOL has an anti-mutagenic activity on the leading strand (73). The notion that PRIMPOL acts as conservator of the genome is supported by the anti-mutagenic activity of PRIMPOL on AID family APOBEC induced mutagenesis in invasive breast cancer. In a genome wide setting, PRIMPOL anti-mutagenic activity on the leading strand on APOBEC dependent mutagenesis, would be expected. In line with this notion, an enrichment of APOBEC mutagenesis was actually found on the leading strand (142, 143).

Using purified PRIMPOL, it was demonstrated that this polymerase/primase is stalled at abasic site under nuclear conditions (70). Therefore, repriming by PRIMPOL has an important anti-mutagenic function. The anti-mutagenic function may be explained by redirecting DDT from error prone TLS to error-free homology-directed template switching. This has also been observed in yeast, where leading/lagging strand primase POLα has been shown to promote recombination directed template switching (144).

We propose a role for PRIMPOL during S phase, despite the observation that AID is mainly active in G1 of the cell cycle, mainly using overexpression settings (24, 25). However, overexpression of AID outside of G1 is toxic to cells, therefore it is not possible to exclude S/G2 activity of AID in overexpression settings (145). Furthermore, U and abasic sites may persist into S phase. In addition, another study suggested that C/G transitions and transversions can occur to some extend during S phase (23).

In conclusion, the G>C over C>G transversion bias is established by the anti-mutagenic activity of PRIMPOL, where PRIMPOL likely reprimes behind abasic sites to stimulate error-free template switching. Strand biases in SHM are established by pro-mutagenic biases like the AID targeting and ncMMR, as well as anti-mutagenic activities, as the G>C over C>G PRIMPOL dependent mutation bias.

## Cell Cycle Regulation of Somatic Hypermutation

AID is active during the G1 phase of the cells cycle (25, 145). Accordingly, G1 is also the cell cycle phase in which the highest levels of Us can be found in the immunoglobulin genes (25). Though, Us and abasic sites may persist into S phases. Furthermore, expression of an AID modified to be specifically expressed in G1 provides all mutations characterizing SHM, whereas AID modified for S/G2/M expression does not support mutagenesis (24). Though, the lack of mutagenesis S/G2/M may be due to the toxicity of overexpressing AID during S phase (145). While most A/T and C/G mutagenesis has been suggested to be limited to G1, C/G transitions and transversions can occur during S phase (23).

During G1, dNTP levels are very low. The low dNTP levels have been shown to impair A/T mutagenesis. Ribonucleotide reductase *Samhd1* deficiency increases the concentration of nucleotides in G1 (146). Surprisingly, this led to a decrease of A/T transversions, but not A/T transitions. However, knocking out *Samdh1* led to increased arrest in late G1, due to high nucleotide levels (147, 148). As AID is active in early G1, the late G1 arrest may affect the SHM profile independently of dNTP levels.

In conclusion, AID is active in G1. A/T and C/G mutations are generated primarily during G1, though a substantial fraction C/G mutations may arise during S phase.

## Conclusion

The discovery of AID laid the foundation in solving the molecular puzzle underlying SHM. Further detailed characterization uncovered distinct mutagenic pathways responsible in generating the typical mutation spectrum of somatically mutated *Ig* genes. Both, pro- and anti-mutagenic activities contribute in establishing defined strand preferences responsible for specific mutation biases recurrently found in these independently generated spectra. Many details regarding the transformation of faithful DNA damage response pathways into effective mutator pathways, often take advantage of error-prone DNA polymerases, as proposed in the Brenner & Milstein model (149), now identified as members of the Y- family of TLS polymerases. At the same time insights from SHM studies fueled general mutation studies and provided novel insights into genome maintenance in general.

## Author Contributions

All authors listed have made a substantial, direct and intellectual contribution to the work, and approved it for publication.

### Conflict of Interest Statement

The authors declare that the research was conducted in the absence of any commercial or financial relationships that could be construed as a potential conflict of interest.
